# Enabling usable science takes a community: Using our roles as funders to catalyze change

**DOI:** 10.1371/journal.pbio.3002675

**Published:** 2024-06-10

**Authors:** Kayla J. Ripple, Charlotte Hudson, Emily Knight, Jason P. Landrum, Victoria Bell, Sarah L. Close

**Affiliations:** Lenfest Ocean Program, The Pew Charitable Trusts, Washington, DC, United States of America; Arizona State University, UNITED STATES

## Abstract

Calls to support co-designed and usable science to inform management of natural resources is growing. This Perspective argues that there is a role for everyone within the usable science community and that, together, we can catalyze positive change.

## Teaser

Calls to support co-designed and usable science to inform management of natural resources are growing. Making the shift will require diverse collaborations between those who hold, share, and use knowledge.

Climate change and other “wicked” problems are rapidly altering the ecological landscape and creating management challenges faster than we can respond. As individuals in the field of marine science philanthropy with the Lenfest Ocean Program, we envision a world where diverse groups of knowledge holders, such as scientists, community leaders, and Indigenous knowledge holders, and decision-makers, including managers, practitioners, and communities, can come together to effectively inform the sustainable management of natural resources. From our experience, user-driven research involving inclusive, collaborative relationships can produce data that is relevant and more reflective of decision-maker needs, leading to a higher likelihood of developing the sustainable, durable management for ecosystems and communities to thrive. Here, we reflect on what we have learned and share our insights with others who can take the work further.

Over the past 20 years, the Lenfest Ocean Program has attempted to merge two well-established, but not well-connected, paths: identifying and supporting credible, legitimate, and salient research [1], and communicating research results to audiences who are in positions to use that information for the public good [2]. Alongside others endeavoring in similar spaces, we have learned that projects with increased engagement between researchers and user groups at the project’s start are often more successful at informing their intended audiences, as opposed to projects that wait to engage user groups until the research results are final [3]. Early on, this motivated us to implement the concepts of co-design [4], co-production [5], and useful and usable science [6] into our grantmaking processes. In parallel, the past decade has brought growing interest from public and private funding organizations to invest in these concepts. Yet, even as the momentum for usable science has grown, real barriers remain. Now, the challenge is how to expand the field so that the capacity exists to work at the scale required by 21st century challenges, and in a way that includes the many different people and communities who use, share, and develop knowledge.

Ultimately, we assert that we need a culture shift in the way science is funded, research is designed, and knowledge is shared and used to catalyze lasting change. To be successful, each member of the usable science community has a unique role (Box 1). At the risk of adding more jargon to a field already rife with terminology (but not wanting to oversimplify the goals, motivations, and needs of individuals or groups), we refer here to actors in the usable science community in broad terms based on their interactions with knowledge. There are those who hold knowledge and are therefore able to share it (knowledge holders), those who use knowledge to inform decisions (knowledge users), and those who support the development of usable knowledge (knowledge supporters). Some individuals may see themselves reflected in more than one group, but the point is that at each stage, there are different ways to facilitate more collaborative and trusting interactions.

**Box 1. Change is happening, but to create lasting impacts, each member of the usable science community has a role to play**

*Knowledge Holders*

- Examples: Scientists, Indigenous Communities, Local Communities, Natural Resource Managers
- Role: Share knowledge that is relevant and key to decision-making.
- Summary: Individual and collective contributions of knowledge holders should inform decision-making, but only when their contributions are valued, protected, and shared equitably. To support this, organizations should recognize and increase capacity for boundary spanners that can act as a neutral conduit for the flow of knowledge.

*Knowledge Users*

- Examples: Policy- and Decision-makers, Tribal Leaders, Natural Resource Managers
- Role: Create pathways to identify and center diverse knowledge types that are used and valued in deliberations.
- Summary: Historic reliance on quantitative data for decision-making has created a barrier for the use of other information sources (e.g., qualitative data and oral histories). Knowledge users should create pathways for including more knowledge types that reflect societal and cultural values.

*Knowledge Supporters*

- Examples: Government Funding Agencies, Philanthropies, Venture Capital, NGOs
- Role: Increase financial investments that support the development of usable science.
- Summary: More private and public funds are needed to enable co-designed research and sustained engagement. Knowledge supporters should tailor their investment strategies, through internal and external practices, to encourage the development of strong collaborations and long-term relationships between groups that have not traditionally worked together.

Knowledge holders comprise a diverse group. Some identify as scientists trained in the Western tradition of knowledge, others hold knowledge stemming from non-Western worldviews, culture, and traditions (e.g., Indigenous knowledge or traditional ecological knowledge), while others identify as individuals who derive knowledge from observations and experiences using natural resources. We believe that knowledge from these varied sources can and should inform decisions, but only when their individual and collective contributions are valued, protected, and shared equitably within decision-making contexts [7]. In this case, we have found that investing in capacity for intermediaries, or boundary spanners, whose role is to facilitate the exchange of information to support decision-making, is key [8]. Boundary-spanning individuals and organizations can add capacity for building trusted relationships between actors who may not have historically worked together. Unlike advocates, who may be bound by political or bureaucratic pressures that can inadvertently push them towards an agenda or timeline, intermediaries can devote the time needed for enabling the flow of knowledge across sectors in a neutral way [9]. Increasing capacity for these activities, whether it be through financial investment or adapting organizational structures to incentivize boundary-spanning activities at academic institutions, non-governmental organizations (NGOs), and government agencies, could profoundly change the way knowledge is created and shared [10].

Knowledge users, such as policymakers, managers, practitioners, rights holders, and stakeholders, aim to leverage existing knowledge to understand all sides of an issue, evaluate tradeoffs associated with possible solutions, and make timely decisions that lead to optimal outcomes for people and the environment. However, the perception that quantitative data is the most credible kind has shaped a hierarchy of what kind of information is considered in decision-making. This perception can cause critical information to be missed that considers the larger picture. Qualitative information and oral histories, for example, often reflect the societal values and cultural significance of natural resources that quantitative information does not capture. Institutional structures rooted in old processes that favored quantitative data may be one barrier to moving forward. A first step could be to make way for and expand the sources of knowledge that are used and valued in deliberations [1,7].

Knowledge supporters may also come in varied forms. Most pertinently, government funding agencies, private philanthropies, business and venture capital providers, and NGOs are in a privileged position to add capacity for the development and sharing of the next generation of usable knowledge, both through external financial support and internal practices. These organizations can contribute towards a more inclusive future through a number of promising grant practices, such as including grant criteria that emphasize collaboration, explicitly valuing peoples’ participation in project activities through direct funding or honoraria, and including project deliverables to incentivize co-designed and co-produced research projects [11]. Furthermore, employing staff as intermediaries that aid grantees in creating feedback loops and relationships with managers and communities can help lay the foundation for these partnerships to persist, even after a project is complete. Ultimately, more public and private research funds must follow suit and help uplift and expand upon these efforts.

Generating science that is useful and benefits nature and society is essential for the durable and sustainable management of natural resources. We believe it is the responsibility of everyone in the natural resource community to create the enabling conditions for science to be impactful in decision-making. To do so will require knowledge holders, users, and supporters to pave pathways that expand how knowledge is generated and used for decision-making. In doing so, they can build a world where diverse perspectives and information more effectively promote durable natural resource management for all.
